# Impact of seven conservation methods on the nutritional value, phytochemical composition, and chemical antioxidant capacity of *Terfezia boudieri* and *Tirmania nivea* from Southeastern Tunisia

**DOI:** 10.3389/fphar.2026.1816928

**Published:** 2026-10-02

**Authors:** Nadia Ghanney, Leila Ben Yahia, Haifa Hlel, Belgacem Lachiheb, Talel Bouhamda, Nahed Fakhfakh, Kamel Nagaz

**Affiliations:** 1 Laboratoire dʼAridoculture et Cultures Oasiennes, Institut des Régions Arides de Médenine, Medenine, Tunisia; 2 Département de Génie Biologique, Institut Supérieur de Biologie Appliquée de Médenine, Medenine, Tunisia; 3 Laboratoire Central, Institut des Régions Arides de Medenine, Medenine, Tunisia

**Keywords:** antioxidant activity, conservation methods, desert truffles, functional nutraceutical potential, micronutrient bioavailability, nutritional profiling, phenolic metabolites

## Abstract

**Introduction:**

Desert truffles (*Terfezia boudieri* and *Tirmania nivea*) are edible fungi valued for their nutritional and nutraceutical properties. However, the effects of post-harvest preservation on their bioactive composition and health-related quality remain poorly understood. This study evaluated the influence of seven conservation methods on the nutritional and functional properties of both species collected from the arid regions of southeastern Tunisia.

**Methods:**

Seven preservation techniques (sun drying, oven drying, deep freezing, lyophilization, chips processing, marination, and olive‐oil preservation) were assessed. Nutritional composition, mineral content, vitamins, carotenoids, phenolic compounds, and antioxidant activity were determined using standard analytical methods. Data were analyzed by two-way ANOVA to evaluate the effects of preservation method, species, and their interaction.

**Results:**

Preservation method was the main source of variation, explaining up to 99.06% of protein, 95.65% of carbohydrate, 94.88% of antioxidant activity, 88.19% of potassium, and 87.07% of total phenolic variability (all P < 0.0001), whereas species effects were comparatively minor. Oven drying and lyophilization best preserved protein content (≈27–28%) and antioxidant activity (87–89% radical scavenging activity). Lyophilization and marination retained the highest mineral concentrations, while lyophilization also preserved the greatest riboflavin (2.69–3.94 ppm) and lycopene (0.15–0.16 ppm) contents. Oven drying produced the highest phenolic acid levels, whereas chips processing consistently reduced proteins, minerals, vitamins, and bioactive compounds.

**Discussion:**

Preservation method is the principal determinant of the nutritional and nutraceutical quality of desert truffles. Oven drying and lyophilization were the most effective strategies for maintaining health‐promoting compounds, supporting their application as optimal post‐harvest technologies to maximize the functional food value and commercial potential of *T. boudieri* and *T. nivea*.

## 1 Introduction

Desert truffles have gained growing scientific attention due to their ecological importance in arid ecosystems, their promising nutraceutical attributes, and their rising economic relevance across North Africa and the Middle East. Recent advances in food chemistry, metabolomics, and fungal genomics have improved understanding of their nutritional composition, bioactive metabolites, and potential for sustainable cultivation (Khalifa et al., 2019; Wang and Marcone, 2011). Studies demonstrate that desert truffles typically contain moderate protein, low fat, essential minerals, and diverse phenolic constituents that contribute to strong antioxidant capacity (Elkhateeb et al., 2024; Guetat et al., 2024; Lee et al., 2020). *Terfezia arenaria*, for example, shows a rich nutritional and aromatic profile, with metabolomics and sensory tools revealing complex volatile and bioactive signatures (Ferreira et al., 2023). UHPLC-MS and UPLC/MS-MS analyses of *Tirmania nivea*/*Terfezia boudieri* and *Tirmania nivea*/*Terfezia claveryi* report respectively gallic acid, catechins, quercetin derivatives phenolics and resveratrol, oleic acid, margaric acid which are linked to antioxidant, antimicrobial, and anti-inflammatory properties (Hammami et al., 2023; Darwish et al., 2021).

Biochemical research confirms that extracts of *T. claveryi*, *T. boudieri*, and *T. nivea* exhibit strong radical-scavenging activity correlated with phenolic and flavonoid content (Al Obaydi et al., 2020; Vahdani et al., 2017). Anti-inflammatory effects have also been documented, notably in *T. boudieri,* which modulates LPS/IFN-γ–induced pathways (Dawood et al., 2023). Metabolomic differentiation reinforces species-specific biochemical variability, with distinct metabolic fingerprints shown across desert truffle taxa (Li et al., 2019). Other reviews emphasize the emerging role of both temperate and desert truffles as promising functional foods and nutraceutical ingredients (Baldelli et al., 2025; Al Ali et al., 2021; Lee et al., 2020; Hamza et al., 2016a; Hamza et al., 2016b).

From an agroecological perspective, interest in desert truffles is rising alongside the need for climate-resilient crops. Genomic studies have identified key genes related to mycorrhizal symbiosis, fruiting, and drought adaptation, providing a foundation for future domestication (Martin and Van der Heijden, 2024). Agronomic assessments highlight the potential of *Terfezia*–*Helianthemum* symbioses to improve soil fertility and support sustainable dryland agriculture (Satish et al., 2022). Ethnoecological work from North Africa and the Arabian Peninsula documents long-standing harvesting traditions and culinary uses, underscoring the cultural and socio-economic value of desert truffles (Alrhmoun et al., 2025).

Despite this expanding interest, *T. boudieri* and *T. nivea* from North Africa remain under-investigated, with few multi-parameter studies examining how conservation techniques affect their nutritional composition, phenolic profiles, and biological activity. Evaluating conservation is essential, as processing is known to influence truffle quality. High-temperature drying (sun or oven) degrades or reduces volatile phenolics, and lowers antioxidant activity (ElGamal et al., 2023; Phong et al., 2022). In contrast, lyophilization and deep freezing consistently provide superior retention of bioactive molecules and sensory characteristics (Babaei Rad et al., 2025; Phong et al., 2022). Novel approaches, such as mild thermal treatment at 63 °C or vinegar-based preservation, offer improved microbial stability but still induce moderate biochemical losses (Alu’datt et al., 2023; Daei et al., 2023). Oil-based conservation, widely used in traditional practices, may protect certain aromatic and lipophilic compounds, though its effects vary across studies.

Despite the growing interest in desert truffles as valuable sources of nutrients and bioactive metabolites, comprehensive studies comparing the impact of different preservation techniques on their nutritional and functional quality remain scarce, particularly for North African species. The high moisture content of fresh truffles (70%–82%) makes them extremely perishable and metabolically active, resulting in rapid deterioration through water loss, respiration, enzymatic reactions, and microbial growth (Ramteke et al., 2020; Dawadi et al., 2022). These characteristics not only limit their shelf life but also complicate compositional analyses, as fresh samples lack a stable dry-weight basis and are difficult to homogenize for chemical extraction. Consequently, freeze-drying and other dehydration techniques are commonly employed prior to analysis to stabilize sensitive metabolites, prevent microbial spoilage, facilitate sample handling, and improve the reproducibility of analytical measurements (Hamza et al., 2016b; Tejedor-Calvo et al., 2020). However, processing and preservation treatments can markedly influence the physicochemical, nutritional, and functional properties of food matrices, thereby affecting their overall quality and stability during storage (Paliwal et al., 2023), thereby influencing the overall nutritional and functional value of products.

Addressing this knowledge gap, the present study systematically evaluates the effects of seven preservation methods; which consist of sun drying, oven drying, deep freezing, lyophilization, frying into chips, marination in vinegar, and preservation in olive oil; on the nutritional composition, biochemical characteristics, and antioxidant properties of *T. boudieri* and *T. nivea* collected from southeastern Tunisia. Through proximate composition analysis, vitamin and mineral determination, LC–MS-based profiling of sugars and phenolics, and antioxidant assays (DPPH, total phenolic content, and total flavonoid content), this work aims to identify the most effective preservation strategies for maintaining the nutritional and functional quality of these desert truffles and to support their sustainable valorization within arid-region agri-food systems.

## 2 Materials and methods

### 2.1 Chemicals and reagents

HPLC-grade solvents were used for all chromatographic analyses. Analytical reagent-grade acetonitrile and methanol were obtained from Lab-Scan (Tedia Company, United States). Ultrapure water for HPLC and sample preparation was produced using a Millipore Simplicity system (Millipore, Molsheim, France). All vitamin standards (water- and fat-soluble) were of chromatography grade and purchased from Sigma Chemical Co. (Poole, Dorset).

### 2.2 Truffle samples

Samples of *T. boudieri* and *T. nivea* (Figure 1), two ascomycetes belonging to the *Terfeziaceae* family, were collected during the January–March harvesting season from two traditional truffle-growing areas in southeastern Tunisia: The Dhahir region (32°28′27.5″N 11°16′03.8″E) of Ben Guerdane and the Th’hba area (32°07′00.6″N 10°44′46.9″) in Tataouine. These sites are situated within the lower arid bioclimatic zone, characterized by an annual mean rainfall of approximately 180 mm. Sampling sites were selected based on guidance from local Saharan inhabitants and nomadic communities, as well as expertise from desert-truffle researchers. The truffle specimens used in this study were identified based on their morphological characteristics and confirmed by IRA Medenine researchers, specially by the mycologist Dr. Imed SBISSI (Sbissi et al., 2011). Taxonomic nomenclature was verified using Index Fungorum (The Royal Botanic Gardens, 2026). The investigated species were identified as *T. boudieri* Chatin (*Pezizaceae*) and *T. nivea* (Desf.) Trappe (*Pezizaceae*). These regions are widely recognized as established natural habitats for both of desert truffles (Bradai et al., 2013).

**FIGURE 1:**
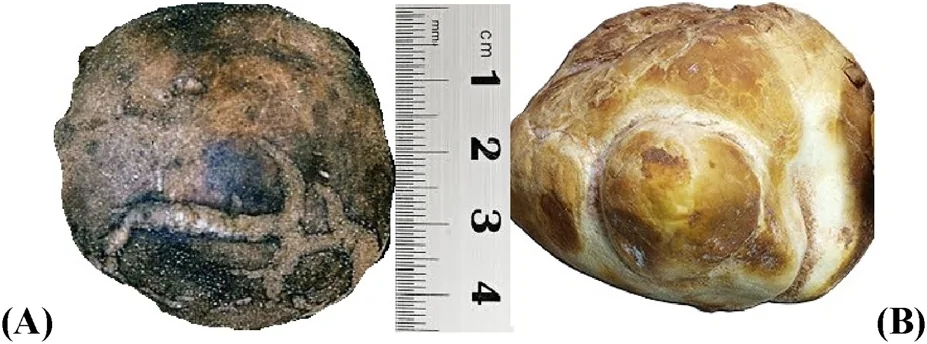
Truffle samples of Terfezia boudieri **(A)** and Tirmania nivea **(B)**. Adapted from photographs available on Ascomycete.org under the Creative Commons Attribution–NonCommercial (CC BY‐NC) license; *T. boudieri* image © P. Roux (Coll. PR 4135).

### 2.3 Sample preparation, conservation treatments, storage conditions, and handling procedures

Fresh fruiting bodies of desert truffles were collected at maturity and transported to the laboratory immediately after harvesting under insulated conditions to minimize temperature fluctuations. Upon arrival, samples were carefully cleaned using sterile distilled water to remove soil particles and external debris, followed by gentle drying with sterile tissue paper. Damaged or deteriorated specimens were excluded from the study. All samples were then sliced into homogeneous strips to ensure uniformity across all experimental treatments.

All sample handling prior to processing (Heinrich et al., 2022) was conducted under controlled laboratory conditions (20 °C–25 °C; relative humidity 50%–60%; atmospheric pressure ≈1 atm). Preparations were performed rapidly to minimize enzymatic degradation, oxidation, and moisture loss. Samples were then randomly assigned to different conservation treatments as described below.

#### 2.3.1 Sun drying

Thin truffle strips were arranged in sterile glass Petri dishes and exposed to direct sunlight under ambient environmental conditions (28 °C–30 °C). Drying was carried out for approximately 7 days, with periodic weighing until a constant mass was achieved.

#### 2.3.2 Oven drying

Thin strips were placed in glass Petri dishes and dehydrated in a vacuum oven at 50 °C for approximately 3 days, until constant weight was reached.

#### 2.3.3 Deep freezing

Fresh truffle samples were vacuum-sealed in food-grade plastic bags and stored at −20 °C for 3 months prior to analysis.

#### 2.3.4 Lyophilization

Representative samples of each species were sliced into thin strips and pre-frozen at −40 °C for 1 h. Freeze-drying was then performed for 72 h at −40 °C using a Freeze Dry System/Lyph Lock 4.5 L (77510-00) operating at a pressure of 1.33 × 10^−3^ mbar. The resulting dried samples were transferred into airtight glass vials and stored for 3 months under dry, dark conditions until biochemical analyses were performed.

#### 2.3.5 Chip conservation

Truffle slices (2–3 mm thickness) were briefly immersed in a vinegar solution, then fried under controlled conditions. The resulting chips were drained on absorbent paper to remove excess oil and stored in a desiccator at room temperature until analysis.

#### 2.3.6 Marination

Truffle pieces were placed in sterile glass containers and immersed in a solution composed of 225 mL sterile water, 25 mL vinegar, 30 g salt, and lemon slices. Samples were completely covered with a layer of olive oil to ensure anaerobic conditions and stored under refrigeration (4 °C ± 1 °C) until analysis.

#### 2.3.7 Preservation in olive oil

Sliced truffles were transferred into sterilized glass jars and completely covered with olive oil, ensuring a 5 mm headspace and absence of trapped air bubbles to minimize oxidation. Samples were stored in sealed containers until biochemical analyses.

### 2.4 General handling and storage conditions

Throughout the experimental workflow, all samples were handled under identical hygienic conditions to ensure comparability of analytical results. After treatment, dried samples were stored in airtight containers protected from light and moisture at room temperature (20 °C–25 °C), while refrigerated treatments were maintained at 4 °C ± 1 °C. All analyses were conducted within a standardized post-processing period to minimize variability associated with storage duration and environmental exposure.

## 3 Nutraceutical analysis

To enable a comprehensive comparison of preservation techniques, the nutraceutical properties of truffle samples subjected to all seven conservation methods were systematically evaluated. Each conserved sample was analyzed for its proximate composition, fiber fractions, mineral and vitamin contents, phenolic and flavonoid profiles, fatty acid composition, and antioxidant activity. This approach allowed us to assess how different storage practices influence the nutritional quality and bioactive potential of *T. boudieri* and *T. nivea*, thereby identifying the most suitable methods for maintaining their functional properties.

### 3.1 Proximate analysis, fibers, minerals, vitamins, and fatty acids

#### 3.1.1 Proximate composition

Dry matter, protein, fat, and ash contents were determined following AOAC (1995) standard procedures. Total nitrogen (N) was measured by the Kjeldahl method, and crude protein was calculated using the conversion factor N × 6.25. Lipid content was assessed via Soxhlet extraction, while ash content was obtained by incinerating samples at 550 °C. Organic matter, total carbohydrates, and caloric value were calculated by difference according to Hamza et al. (2016a).

#### 3.1.2 Fiber determination

Dietary fiber fractions were quantified using the Neutral Detergent Fiber (NDF) and Acid Detergent Fiber (ADF) protocols described by ANKOM Technology (2000), allowing for accurate estimation of structural carbohydrate components in the samples.

#### 3.1.3 Sugars analysis

Sugar profiling was carried out using a Shimadzu UFLC XR system equipped with a refractive index detector (RID-10A) and a NH_2_ column (3 μm, 150 mm × 3.0 mm). Chromatographic separation was conducted isocratically at 40 °C using a mobile phase of water/acetonitrile (15:85, v/v) at a flow rate of 0.40 mL·min^−1^ and a 5 µL injection volume. Identification of sugars was based on retention time matching with analytical standards, and quantification was performed using external calibration curves; results were expressed in ppm.

#### 3.1.4 Mineral analysis

Samples were air-dried, further dried at 105 °C, and ground to a fine powder. One gram of material was digested following Akyuz (2013) and Akyuz and Kirbag (2010) using a mixture of HNO_3_, H_2_SO_4_, and H_2_O_2_ (8:1:1 v/v), heated at 100 °C for 10–15 min. After cooling, the digest was diluted to 50 mL with deionized water and filtered, with all equipment thoroughly rinsed to avoid contamination. Trace elements (Fe, Zn, Mn, Cu, Cr, Cd, Co, Ni, Pb) were quantified using Atomic Absorption Spectrometry (AAS), while macro elements (K, Ca, Na) were measured by Atomic Emission Spectrometry (AES), following AOAC (1990) recommendations.

#### 3.1.5 Vitamin analysis

Fat- and water-soluble vitamins were analyzed using a Shimadzu UFLC XR system coupled to a quadrupole 2020 mass detector and fitted with a C18 Aquacil column (3 μm, 150 mm × 3.0 mm). Separation was performed at 40 °C with a 5 µL injection and a 0.4 mL·min^−1^ flow rate.

For fat-soluble vitamins, the mobile phase consisted of water (5 mmol ammonium formate, 0.1% formic acid) and methanol (0.1% formic acid), using a gradient from 70% to 100% methanol over 15 min, maintained until 39 min, followed by re-equilibration. Detection was achieved by APCI with optimized source parameters.

For water-soluble vitamins, the mobile phase included water and methanol each containing 10 mmol ammonium formate and 0.1% formic acid. A gradient from 2% to 55% methanol over 12 min, then to 100% by 15 min, was applied before returning to initial conditions. Detection was performed using ESI under established conditions. In both cases, vitamin identification relied on comparison with certified reference standards.

### 3.2 Polyphenols, flavonoids, phenolic acids, and antioxidant capacity

#### 3.2.1 Total phenolic and flavonoid contents

Total phenolic content (TPC) in methanolic extracts of *T. boudieri* and *T. nivea* was measured using the Folin–Ciocalteu assay (Dewanto et al., 2002). Extracts (0.5 mL) were mixed with Folin–Ciocalteu reagent and Na_2_CO_3_, incubated for 30 min in the dark, and absorbance read at 765 nm, with results expressed as mg GAE·g^−1^ DW. Total flavonoid content (TFC) was determined by the AlCl_3_ colorimetric method, measuring absorbance at 510 nm and expressing results as mg CE·g^−1^ DW.

#### 3.2.2 Phenolic acids and flavonoids

Phenolic acids were quantified using a Shimadzu UFLC XR system equipped with an Inertsil ODS-4 C18 column (3 μm, 150 mm × 3.0 mm) at 40 °C, with a 20 µL injection and 0.5 mL·min^−1^ flow rate. The mobile phase consisted of water/methanol/acetic acid (A) and acetonitrile/water/acetic acid (B), using a linear gradient from 10% to 100% B over 50 min. Detection was carried out by LC–ESI–MS, and metabolites were identified using authenticated standards (LGC, Sigma-Aldrich).

#### 3.2.3 Antioxidant capacity (DPPH assay)

Antioxidant capacity was evaluated using the DPPH radical scavenging assay as described by Brand-Williams et al. (1995). Extracts (1 mL) were mixed with 2 mL of 0.1 mM DPPH solution, incubated for 30 min in the dark, and absorbance measured at 517 nm. Radical Scavenging Capacity (RSC) was calculated using control and sample absorbances (A_0_ and A_s_), and results were expressed as inhibition percentage.

DPPH radical scavenging capacity (%) was calculated as:

$$\text{RSC }\left(\%\right)=\left[\left(A_{0}-A_{S}\right)/A_{0}\right]\times 100,$$

where *A*_*0*_ is the absorbance of the DPPH control solution, and *A*_*S*_ is the absorbance of DPPH solution containing the extract or standard.

### 3.3 Statistical analysis

Results were presented as mean ± standard deviation (SD). Statistical analyses were performed using SPSS software, while graphical representations were generated with GraphPad Prism 5. Differences among treatments were evaluated by analysis of variance (ANOVA), and the effects of species and conservation method, as well as their interaction, were assessed using two-way ANOVA. Statistical significance was established at p < 0.05. When relevant, exact p-values or more stringent significance levels (e.g., p < 0.01 or p < 0.0001) are reported to indicate the strength of the statistical evidence.

## 4 Results

### 4.1 Proximate components

The proximate composition of both truffle species was mainly governed by the conservation method, which represented the principal source of variation across most parameters, while species identity and method × species interactions contributed to a lesser but still significant extent.

These findings highlight preservation technique as the key determinant shaping nutritional composition and justify the detailed results presented thereafter (Figure 2).

**FIGURE 2:**
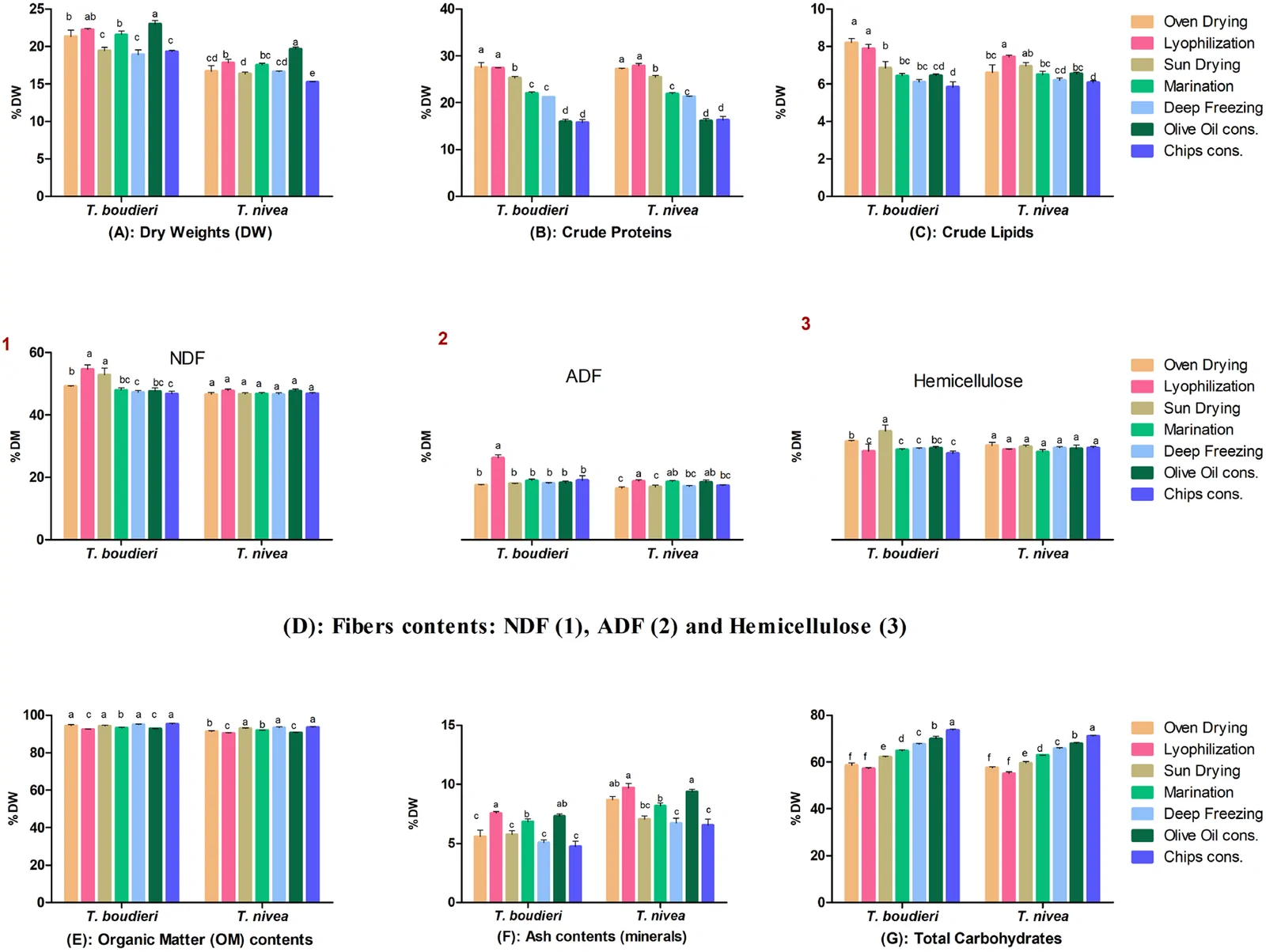
Proximate components (Dry weights **(A)**, Crude protein **(B)**, Crude lipids **(C)**, Fibers **(D)**, Organic matter **(E)**, Ash **(F)** and total carbohydrates **(G)**) in *Terfezia boudieri* and *Tirmania nivea* after seven conservation methods.

#### 4.1.1 Dry weights

Dry weight (Figure 2A) was significantly affected by both truffle species and conservation method, with species explaining most of the variability (62.34%), followed by conservation method (32.37%), and a smaller but significant interaction (2.73%). Overall, *Terfezia boudieri* consistently showed higher dry weight values than *Tirmania nivea* across all treatments (≈18.9–23.0% vs. 15.3%–19.6%). In both species, lyophilization produced the highest dry weights (22.25% in *T. boudieri*; 17.82% in *T. nivea*), closely followed by olive oil conservation (23.0% and 19.64%, respectively), whereas sun drying and deep freezing resulted in lower and often similar values (≈19.4–18.9% in *T. boudieri*; ≈16.3–16.7% in *T. nivea*). Oven drying significantly increased dry weight in *T. boudieri* (21.32%) but had a limited effect in *T. nivea* (16.69%). Chips consistently showed the lowest dry weights (19.31% and 15.26%). The significant interaction reflects a greater sensitivity of *T. boudieri* to conservation method, while *T. nivea* exhibited more moderate variations across treatments.

These differences reflect the known impacts of preservation on water balance and tissue structure. Dehydration methods (lyophilization, oven drying) efficiently remove water, concentrating solids and yielding higher dry weights, particularly in *T. boudieri*. Liquid- or fat-based treatments (olive oil conservation, marination) alter dry matter through limited dehydration combined with uptake of exogenous components, which can increase measured dry weight. Deep freezing largely retains water but disrupts cells via ice crystals, leading to solute redistribution or loss after thawing and dry weights similar to or lower than sun drying. Chips processing, combining heat and fragmentation, promotes solid losses and results in the lowest dry matter. Overall, preservation reshapes the water–solid equilibrium and tissue microstructure, with species-specific traits modulating the magnitude of these effects.

#### 4.1.2 Crude protein

Crude protein content (Figure 2B) was overwhelmingly influenced by the conservation method, which explained 99.06% of total variation and was highly significant (p < 0.0001), Mean protein values indicated that oven drying (≈27.2–27.6%) and lyophilization (≈27.4–27.8%) produced the highest levels with no significant difference between them (p > 0.05). Sun drying yielded intermediate values (≈25.3–25.4%) that were significantly higher than freezing and preservation treatments (p < 0.001). Deep freezing (≈21.2–21.3%) and marination (≈21.9–22.0%) were statistically similar (p > 0.05) and showed moderate protein retention. The lowest contents were observed for chips conservation (≈15.8–16.3%) and olive oil preservation (≈16.0–16.1%), which did not differ significantly from each other but were significantly lower than all other methods (p < 0.001). Overall, drying techniques, especially oven drying and lyophilization, were the most effective for preserving protein levels.

#### 4.1.3 Crude fat

Crude fat content (Figure 2C) was significantly influenced by conservation method, truffle species, and, importantly, by their interaction, indicating that lipid retention depends not only on processing but also on species-specific responses. The conservation method was the dominant factor, explaining 72.06% of total variability, while the significant interaction (19.02%) highlights contrasting behaviors of *Terfezia boudieri* and *Tirmania nivea* under certain treatments. Mean values showed that for T. boudieri, oven drying produced the highest fat level (8.21%), closely followed by lyophilization (7.89%), while the lowest values occurred with chips preservation (5.84%) and deep freezing (6.11%). In T. nivea, the highest fat content was observed after lyophilization (7.45%), whereas oven drying yielded only 6.60%, demonstrating a species-dependent response. Sun drying gave intermediate values in both species (≈6.85–6.95%). Post-hoc comparisons confirmed that lyophilization generally resulted in significantly higher fat levels than freezing, chips, marination, or oil preservation (p < 0.001), whereas freezing, marination, and oil conservation were often statistically similar. Overall, the results indicate that method choice is the primary determinant of crude fat retention, but its effect varies between species, highlighting a significant method and species interaction.

#### 4.1.4 Fibers

As a whole, the fiber analyses (Figure 2D) indicate that total cell wall material (NDF), its more recalcitrant fraction (ADF), and the derived hemicellulose fraction (NDF–ADF) are all significantly influenced by conservation method and species, with strong interaction effects demonstrating species-dependent responses to processing. Two-way ANOVA results showed that fiber fractions were primarily influenced by the conservation method across all parameters, explaining 37.70% of variation for NDF, 54.04% for ADF, and 49.60% for hemicellulose (all p < 0.0001), with significant species and interaction effects indicating method-dependent species responses. *T. boudieri* consistently exhibited higher fiber values than *T. nivea*, reaching maxima under lyophilization for NDF (54.65%) and ADF (26.25%) and under sun drying for hemicellulose (34.82%), whereas its lowest values generally occurred with freezing or chips treatments (∼46.72–47.22% NDF; ∼17.50–19.02% ADF; ∼27.70–29.35% hemicellulose). In contrast, *T. nivea* showed relatively stable levels across methods (≈46.55–47.72% NDF, 16.42%–18.62% ADF, 28.10%–30.15% hemicellulose) with few significant pairwise differences. Overall, preservation technique is the dominant determinant of fiber composition, with strong responsiveness in *T. boudieri* but marked stability in *T. nivea*.

#### 4.1.5 Organic matter (OM)

Organic matter (OM) content (Figure 2E) was significantly affected by truffle species, conservation method, and their interaction (two-way ANOVA, *P* < 0.01), with conservation method explaining the largest proportion of variance (52.04%), followed by species (40.94%). Overall, *Terfezia boudieri* exhibited higher OM values than *Tirmania nivea* across all conservation methods. In *T. boudieri*, OM content ranged from 92.43% under lyophilization to 95.27% in chips conservation, with intermediate values recorded for sun drying (94.26%), oven drying (94.40%), deep freezing (94.94%), marination (93.17%), and olive oil conservation (92.70%). In *T. nivea*, the lowest OM values were observed following lyophilization (90.29%) and olive oil conservation (90.62%), whereas chips conservation (93.46%) and deep freezing (93.29%) resulted in the highest values; sun drying (92.95%), oven drying (91.31%), and marination (91.83%) showed intermediate levels. Bonferroni *post hoc* comparisons confirmed significant differences among most conservation methods (P < 0.05), with lyophilization and olive oil conservation consistently associated with reduced OM, while chips conservation yielded the highest OM content in both species.

OM exhibited a clear inverse pattern relative to ash content across both species and all conservation methods, reflecting the complementary relationship between mineral matter and organic constituents, with treatments that increased ash (lyophilization and olive oil conservation) consistently associated with reduced OM levels.

#### 4.1.6 Ash contents

Ash content (Figure 2F) was significantly affected by truffle species, conservation method, and their interaction (two-way ANOVA, *P* < 0.01), with conservation method explaining the greatest proportion of variability (52.04%), followed by species (40.94%). Overall, *Tirmania nivea* exhibited higher ash contents than *Terfezia boudieri* under all conservation methods. In *T. boudieri*, ash values ranged from 4.73 g/100 g DW in chips conservation and 5.06 g/100 g DW after deep freezing to 7.57 g/100 g DW following lyophilization, with intermediate values observed for sun drying (5.74 g/100 g DW), oven drying (5.60 g/100 g DW), marination (6.83 g/100 g DW), and olive oil conservation (7.30 g/100 g DW). Similarly, in *T. nivea*, the lowest ash contents were recorded in chips (6.54 g/100 g DW) and deep freezing (6.71 g/100 g DW), whereas lyophilization (9.71 g/100 g DW) and olive oil conservation (9.38 g/100 g DW) resulted in the highest values; sun drying (7.05 g/100 g DW), oven drying (8.69 g/100 g DW), and marination (8.17 g/100 g DW) showed intermediate levels. Bonferroni *post hoc* tests confirmed significant differences among most conservation methods (P < 0.05), with particularly pronounced contrasts between lyophilization and chips or deep freezing in both species.

#### 4.1.7 Total carbohydrates

Carbohydrates content (Figure 2G) was significantly influenced by truffle species and conservation method (two-way ANOVA, *P* < 0.0001), while their interaction was not significant (*P* = 0.4312). Conservation method accounted for the majority of the observed variability (95.65%), followed by species (3.44%). Overall, *T. boudieri* exhibited slightly higher carbohydrate levels than *T. nivea* across all treatments. In *T. boudieri*, carbohydrate content ranged from 57.21 g/100 g DW under lyophilization and 58.65 g/100 g DW after oven drying to 73.66 g/100 g DW in chips conservation, with intermediate values recorded for sun drying (62.15 g/100 g DW), marination (64.75 g/100 g DW), deep freezing (67.65 g/100 g DW), and olive oil conservation (69.93 g/100 g DW). Similarly, in *T. nivea*, the lowest carbohydrate contents were observed following lyophilization (55.01 g/100 g DW) and oven drying (57.47 g/100 g DW), whereas chips conservation resulted in the highest value (71.06 g/100 g DW), followed by olive oil conservation (67.81 g/100 g DW) and deep freezing (65.78 g/100 g DW). Bonferroni *post hoc* tests confirmed significant differences among all conservation methods (P < 0.001), with chips conservation consistently associated with the greatest carbohydrate retention and lyophilization with the lowest levels in both species.

### 4.2 Micronutrient profile

#### 4.2.1 Minerals

Mineral contents (Figure 3) in *T. boudieri* and *T. nivea* reveal that both species identity and conservation method influence mineral retention, with their relative impact varying by element. Sodium (Na) is strongly affected by species (43.79%), method (41.55%), and their interaction (11.43%) (all p < 0.0001); *T. boudieri* shows higher Na than *T. nivea*, with maxima under marination (5,085 vs. 4843 ppm) and lyophilization (4,719 vs. 2,756 ppm), and minima in chips (3235; 2810 ppm) and sun drying or freezing (∼3,685 ppm; 2081 ppm). Potassium (K) is overwhelmingly determined by method (88.19%, p < 0.0001), with smaller contributions from species (7.04%) and interaction (2.10%); highest levels occur under lyophilization (9,491; 8,365 ppm) and olive oil preservation (9,491; 8,365 ppm), lowest in chips (5,581; 4,095) and sun drying (6182; 5782 ppm). Calcium (Ca) depends mainly on method (74.55%, p < 0.0001) and interaction (19.65%), with lyophilization and olive oil yielding the highest Ca (7523; 8082 ppm) and chips or sun drying the lowest (5,793–6,280 ppm; 5,603–5,772 ppm). Iron (Fe) is mostly species-dependent (80.02%, p < 0.0001), with *T. nivea* far higher (1,283–1,600 ppm) than *T. boudieri* (452–664 ppm), while lyophilization and marination increase Fe (*T. boudieri*: 631–664 ppm; *T. nivea*: 1,449 ppm–1,600 ppm) and chips reduce it (415 ppm; 750 ppm). Magnesium (Mg) is shaped primarily by method (71.87%) and species (18.95%), with *T. nivea* higher (586–969 vs. 407–826 ppm); lyophilization and marination maximize Mg (*T. boudieri*: 780–826; *T. nivea*: 918–969 ppm), oven drying and sun drying are intermediate (407–460; 586–753 ppm), and chips reduce it (*T. boudieri*: 407 ppm; *T. nivea*: 490 ppm). Zinc (Zn) is dominated by method (92.89%), with smaller species (3.09%) and interaction effects (1.77%); lyophilization and marination produce the highest Zn (*T. boudieri*: 96–184; *T. nivea*: 115–179), sun/oven drying moderate (67–81; 90–98 ppm), and chips lowest (≈68; 69 ppm). Manganese (Mn) is primarily method-dependent (67.57%), with minimal species or interaction effects; sun drying preserves the highest Mn (5.25; 7.56 ppm), whereas oven drying, freezing, lyophilization, chips, marination, and olive oil reduce it (*T. boudieri*: 2–4.7 ppm; *T. nivea*: 4–6.9 ppm). Overall, conservation method is the main determinant of mineral retention, species dominates for Na, Fe, and Mg, and interactions are element-specific, highlighting that marination and lyophilization generally maximize mineral content while chips preservation and sun drying often yield lower levels.

**FIGURE 3:**
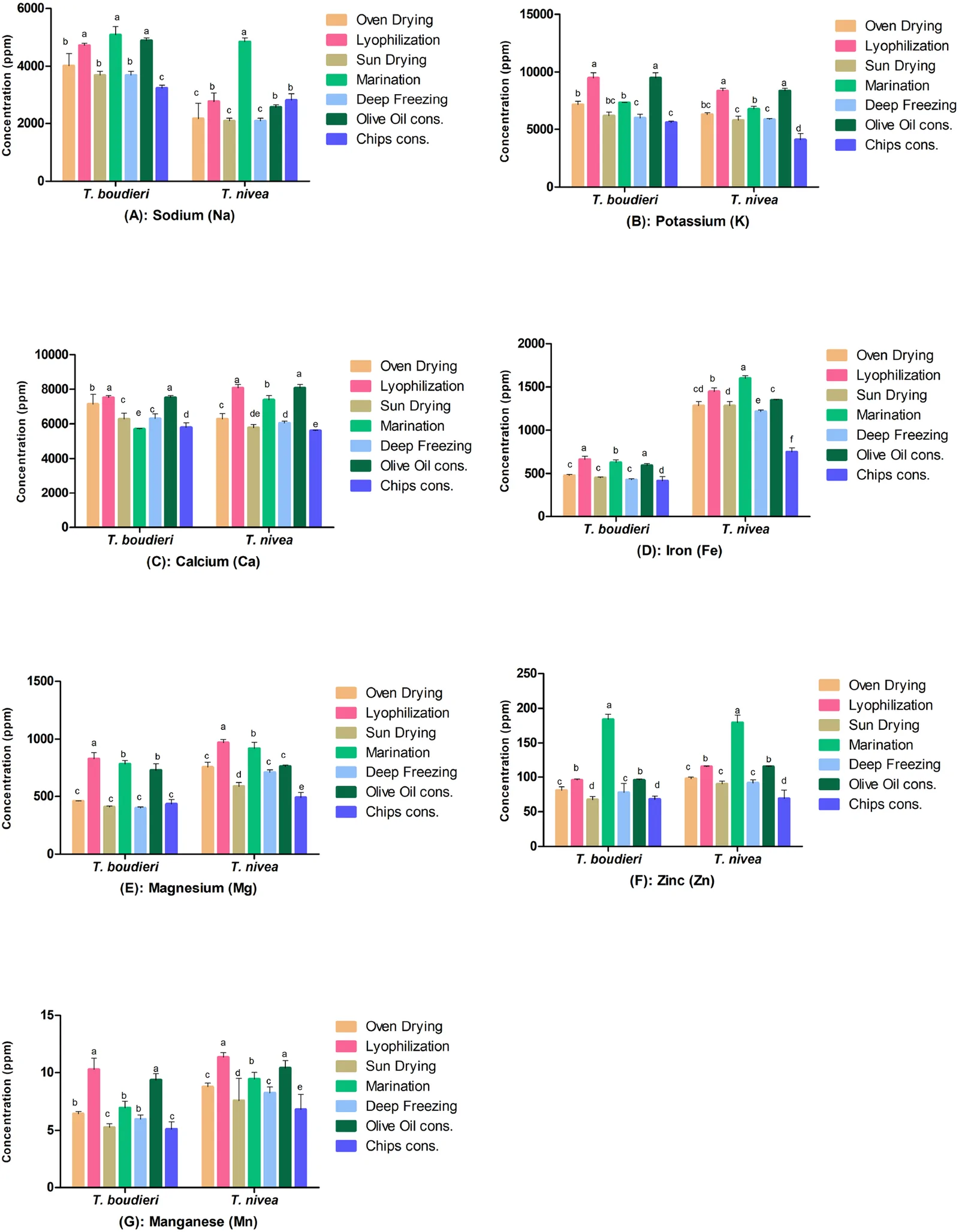
Minerals (Sodium **(A)**, Potassium **(B)**, Calcium **(C)**, Iron **(D)**, Magnesium **(E)**, Zinc **(F)** and Manganes **(G)**) in *Terfezia boudieri* and *Tirmania nivea* after seven conservation methods.

#### 4.2.2 Vitamins

Vitamin levels (Figure 4) in *T. boudieri* and *T. nivea* were primarily influenced by conservation method rather than species, as shown by two-way ANOVA results across lycopene, β-carotene, riboflavin, and tocopherol. While species effects were generally limited, some significant interactions indicated method-dependent responses. Overall, preservation technique emerged as the key factor determining vitamin retention and nutritional quality in both truffle species.

**FIGURE 4:**
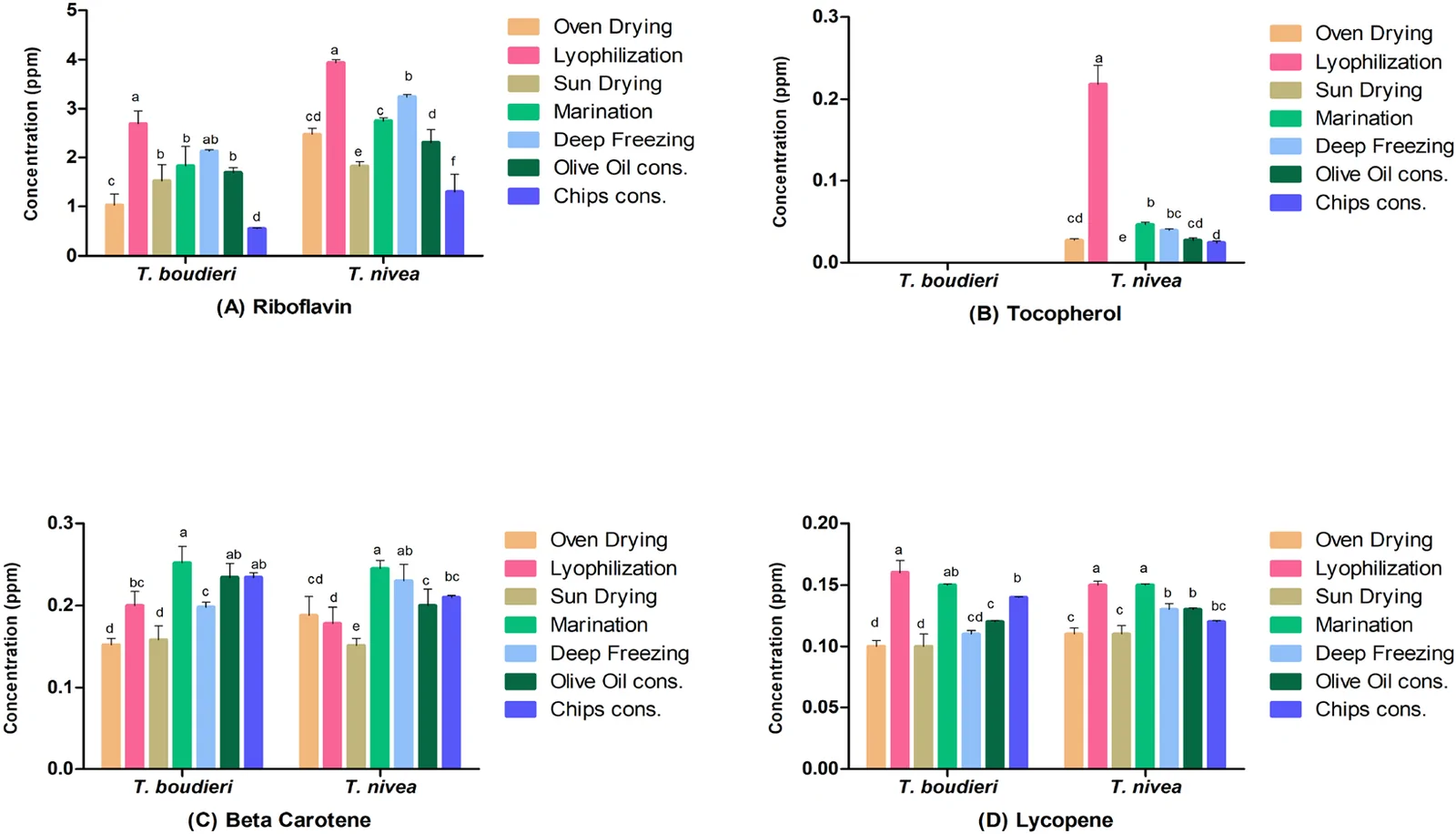
Vitamins (Riboflavin **(A)**, Tocopherol **(B)**, Beta carotene **(C)** and Lycopene **(D)**) in *Terfezia boudieri* and *Tirmania nivea* after seven conservation methods.

##### 4.2.2.1 Riboflavin

The riboflavin content (Figure 4A) in *T. boudieri* and *T. nivea* is strongly affected by the conservation method. In *T. boudieri*, lyophilization gives the highest riboflavin (2.693 ppm), followed by deep freezing (2.125 ppm), sun drying (1.527 ppm), oven drying (1.031 ppm), marination (1.829 ppm), olive oil conservation (1.690 ppm), and chips (0.557 ppm). In *T. nivea*, lyophilization also leads to the highest levels (3.937 ppm), followed by deep freezing (3.240 ppm), oven drying (2.483 ppm), sun drying (1.821 ppm), marination (2.751 ppm), olive oil conservation (2.307 ppm), and chips (1.301 ppm). Lyophilization and deep freezing significantly retain riboflavin compared to drying or chips, while chips consistently result in the lowest values, and *T. nivea* generally contains more riboflavin than *T. boudieri* across all preservation methods.

##### 4.2.2.2 Tocopherol

Tocopherol content (Figure 4B) in *T. boudieri* and *T. nivea* is highly influenced by the conservation method and species. In *T. boudieri*, tocopherol remains consistently very low across all methods (0.0 ppm), showing no significant differences between sun drying, oven drying, deep freezing, lyophilization, chips, marination, or olive oil conservation. In contrast, *T. nivea* shows a marked increase with lyophilization (0.218 ppm), followed by marination (0.046 ppm), deep freezing (0.039 ppm), oven drying and olive oil (0.027 ppm), and chips (0.024 ppm), while sun drying yields 0 ppm. Lyophilization in *T. nivea* significantly preserves tocopherol compared to all other methods (P < 0.001), while methods like sun drying result in the lowest levels, highlighting a strong species -conservation method interaction.

##### 4.2.2.3 Beta-carotene

Beta-carotene content (Figure 4C) in truffles is strongly influenced by the conservation method, with species having no significant effect (P = 0.44) and a modest but significant interaction (13.12%, P = 0.0018). Two-way ANOVA shows that conservation method explains 73.81% of the total variation (P < 0.0001). In general, sun drying yields the lowest beta-carotene levels (0.151–0.158 ppm), while marination (0.245–0.252 ppm) and chips conservation (0.210–0.234 ppm) preserve the highest amounts. Deep freezing (0.198–0.230 ppm), lyophilization (0.178–0.200 ppm), oven drying (0.152–0.188 ppm), and olive oil conservation (0.200–0.234 ppm) provide intermediate levels. Post-hoc comparisons confirm that marination and chips generally result in significantly higher beta-carotene compared to sun drying or oven drying, particularly in *T. boudieri*, while some differences in *T. nivea* are also significant (e.g., deep freezing vs. sun drying, P < 0.001). Overall, conservation method is the key determinant of beta-carotene preservation, with species differences being minimal.

##### 4.2.2.4 Lycopene

Lycopene content (Figure 4D) in truffles is mainly influenced by the conservation method rather than species, as two-way ANOVA shows a significant effect of conservation method (84.92% of variation, P < 0.0001) and interaction (10.36%, P < 0.0001), while species effect is not significant (P = 0.0741). In both *T. boudieri* and *T. nivea*, sun drying results in the lowest lycopene (0.100–0.110 ppm), whereas lyophilization consistently preserves the highest levels (0.160 ppm in *T. boudieri*, 0.150 ppm in *T. nivea*). Other methods such as marination (0.150/0.150 ppm), chips (0.140/0.120 ppm), deep freezing (0.110/0.130 ppm), oven drying (0.100/0.110 ppm), and olive oil conservation (0.120/0.130 ppm) show intermediate values. Post-hoc comparisons indicate that lyophilization significantly increases lycopene relative to sun drying, oven drying, and most other methods (P < 0.001), while differences between other conservation methods are smaller but still often significant, particularly in *T. boudieri*. Overall, lyophilization and marination are the most effective at preserving lycopene, with species differences being minimal.

#### 4.2.3 Sugars

Sugar composition (Figure 5) in *T. boudieri* and *T. nivea* was markedly affected by both conservation method and species, with significant interactions indicating species-specific responses to processing. For glucose, conservation method was the dominant factor, while lactose variation was jointly explained by method, species, and their interaction. Overall, preservation technique emerged as the primary determinant of sugar retention, though species identity played a more pronounced role than observed for vitamins, particularly for lactose.

**FIGURE 5:**
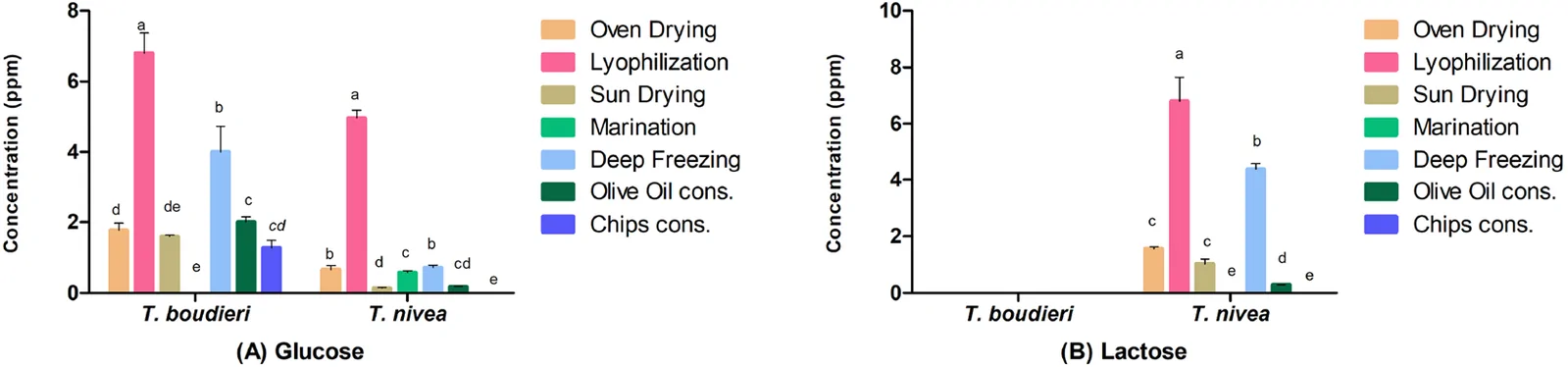
Sugars (Glucose **(A)** and Lactose **(B)**) in *Terfezia boudieri* and *Tirmania nivea* after seven conservation methods.

##### 4.2.3.1 Glucose

Glucose content in truffles (Figure 5A) is markedly affected by both the preservation method and the species, with a highly significant interaction between these factors (P < 0.0001). Among them, the conservation technique plays the dominant role, explaining 78.7% of the variation, while species differences account for 13.1%. Lyophilization proves to be the most effective method for retaining glucose, yielding the highest levels in both *T. boudieri* (6,800 ppm) and *T. nivea* (4,967 ppm). Deep freezing also preserves relatively high glucose concentrations, particularly in *T. boudieri* (4,012 ppm), whereas sun and oven drying result in substantially lower values. Marination and chips processing markedly decrease glucose in *T. boudieri* but only moderately affect *T. nivea*, while olive-oil preservation produces intermediate levels in both species. Post-hoc comparisons confirm that lyophilization and deep freezing generally maintain significantly higher glucose than other methods, especially for *T. boudieri*, emphasizing that both preservation strategy and species identity are key determinants of glucose retention.

##### 4.2.3.2 Lactose

Lactose content (Figure 5B) in truffles is significantly influenced by conservation method, truffle species, and their interaction (all P < 0.0001), with the interaction and conservation method each explaining 36.77% of total variation and species 25.54%. For *T. boudieri*, lactose remains at 0 ppm across all conservation methods, showing no significant differences. In contrast, *T. nivea* shows strong variation: sun drying (1,033 ppm), oven drying (1,573 ppm), deep freezing (4,403 ppm), lyophilization (6,801 ppm), chips (0 ppm), marination (0 ppm), and olive oil conservation (287 ppm). Bonferroni post-hoc tests confirm that lyophilization and deep freezing significantly increase lactose in *T. nivea* compared with sun or oven drying (P < 0.001), while chips, marination, and olive oil conservation significantly reduce it (P < 0.01 to P < 0.001), highlighting both species dependence and strong sensitivity to the preservation method.

### 4.3 Quantitative and qualitative phytochemicals metabolites

#### 4.3.1 Quantitative bioactive metabolites

Quantitative bioactive metabolites (Figure 6) in *T. boudieri* and *T. nivea* were predominantly shaped by conservation method rather than species, as evidenced by two-way ANOVA results for total phenolics, flavonoids, and antioxidant capacity. In all cases, processing technique explained the overwhelming proportion of variation, while species effects were minor but sometimes significant, with interactions indicating method-dependent responses. Similar effects of drying techniques on functional properties and bioactive metabolite stability have been reported in food matrices, particularly under vacuum and through-flow drying conditions, which influence color degradation kinetics and essential oil composition (Neha Sharma and Mohite, 2025). Overall, preservation strategy emerged as the principal factor controlling the retention of phenolics and antioxidant potential in both truffle species.

**FIGURE 6:**
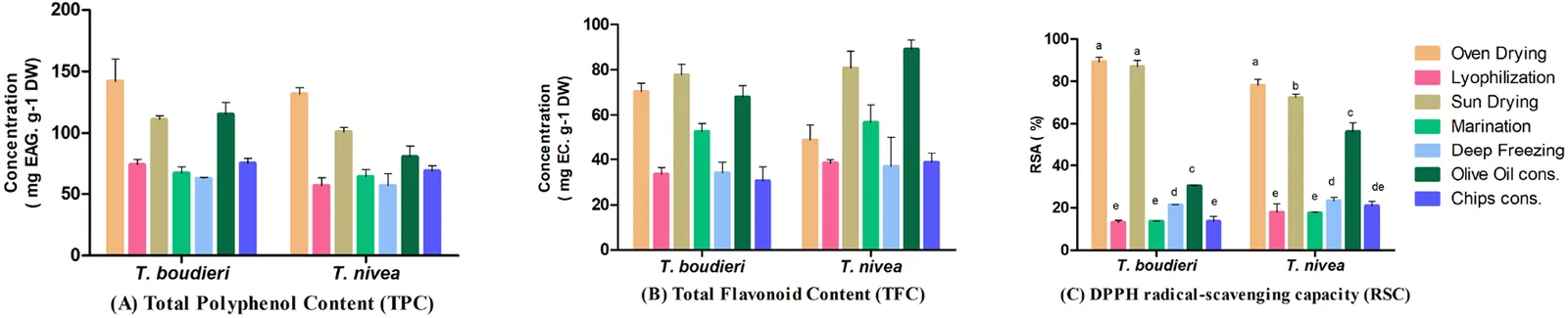
Quantitative bioactive compounds (TPC **(A)**, TFC **(B)** and RSA **(C)**) in *Terfezia boudieri* and *Tirmania nivea* after seven conservation methods.

##### 4.3.1.1 Total polyphenols content (TPC)

The total phenolic content (TPC) was strongly influenced by conservation method, which accounted for the vast majority of total variation (87.07%, P < 0.0001), while truffle species (4.91%, P < 0.0001) and the interaction between species and method (3.19%, P = 0.0191) had smaller but significant effects. Overall, oven drying produced the highest TPC, with values of approximately 142 mg GAE/g for *T. boudieri* and 132 mg GAE/g for *T. nivea*, significantly exceeding all other treatments (P < 0.001). Sun drying showed intermediate levels (≈111 and 101 mg GAE/g, respectively), while deep freezing, lyophilization, chips, and marination resulted in lower and generally similar contents (≈63–74 mg GAE/g). Olive-oil conservation preserved comparatively high TPC, especially in *T. boudieri* (≈115 mg GAE/g), surpassing several non-drying methods. Pairwise comparisons confirmed that processing technique is the dominant determinant of phenolic retention, with drying methods enhancing TPC and non-drying methods tending to reduce it, although the magnitude of these effects varies slightly between species.

##### 4.3.1.2 Total flavonoids content (TFC)

The flavonoid content (TFC), a major subgroup of polyphenols, was predominantly determined by conservation method, which explained 84.22% of total variation (P < 0.0001), whereas species had little effect (0.66%, P = 0.0984), although their interaction was significant (8.81%, P = 0.0002), reflecting method-dependent species responses. Overall, sun drying and olive-oil conservation yielded the highest TFC, reaching approximately 78–81 mg QE/g for *T. boudieri* and *T. nivea*, while deep freezing, lyophilization, and chips preservation produced the lowest and statistically similar values (≈30–38 mg QE/g). Oven drying showed intermediate, species-dependent results, particularly lower levels in *T. nivea* (≈49 mg QE/g). Post-hoc comparisons confirmed that most drying or oil-based treatments significantly exceeded freezing or chips (P < 0.001), demonstrating that preservation technique is the principal determinant of flavonoid retention, with minor species influence but notable interaction effects.

#### 4.3.2 Chemical antioxidant capacity assessed by DPPH Radical-Scavenging Capacity (RSC)

To complement the phytochemical characterization of the truffle samples, their radical-scavenging capacity was assessed using the DPPH chemical assay. This assay provides analytical measures of the ability of sample constituents to quench free radicals under controlled *in vitro* conditions and do not directly reflect pharmacological or *in vivo* antioxidant effects. The DPPH radical-scavenging capacity was almost entirely governed by conservation method, which accounted for 94.88% of total variation (P < 0.0001), while species (0.21%, P = 0.0006) and interaction effects (4.52%, P < 0.0001) were statistically significant but comparatively minor. Overall, the highest radical-scavinging capacity was observed under oven drying and sun drying (≈87–89% in *T. boudieri*; ≈72–78% in *T. nivea*), whereas lyophilization, chips, and marination produced the lowest values (≈13–21%), and deep freezing showed similarly low levels (≈21–23%). Olive-oil preservation yielded intermediate activity, particularly higher in *T. nivea* (≈56%) than in *T. boudieri* (≈30%). Post-hoc comparisons confirmed that drying treatments consistently produced significantly greater antioxidant capacity than freezing or processed forms, demonstrating that preservation method is the dominant determinant of DPPH capacity, with only slight species influence but significant method-dependent responses.

#### 4.3.3 Qualitative bioactive metabolites

Qualitative bioactive profiles in *T. boudieri* and *T. nivea*, including phenolic acids and flavonoids, were significantly influenced by both conservation method and species, with strong interaction effects indicating species-specific responses to processing. Overall, conservation technique was the dominant determinant of metabolite composition, while species differences played a secondary yet notable role, particularly for flavonoids. These findings underscore the importance of selecting appropriate preservation methods to maintain the qualitative phytochemical integrity of desert truffles.

##### 4.3.3.1 Phenolic acids (Pa)

Phenolic acids (Quinic, Gallic, Protocatchuic, Chlorogenic, 4-O-caffeoylquinic, p-coumaric, trans frulic, 3,4-di-O-caffeoyquinic and Rosmarinic acids) in truffles (Figure 7) showed that the conservation method was the main factor affecting their content (73.47% of total variation, P < 0.0001), followed by the interaction between method and species (20.73%, P < 0.0001) and the truffle species alone (5.75%, P < 0.0001). Bonferroni post-hoc tests revealed that for *T. boudieri*, sun drying (383.4) and oven drying (605.4) resulted in the highest phenolic levels, with all other methods significantly lower (e.g., lyophilization 156.8, chips 112.4, marination 173.5, olive oil 229.9; P < 0.001). For *T. nivea*, sun drying (262.7) and oven drying (305.1) were also superior, while marination (338.4) preserved phenolics better than deep freezing (96.46), lyophilization (124.7), chips (42.92), or olive oil (195.2), highlighting a significant species method interaction.

**FIGURE 7:**
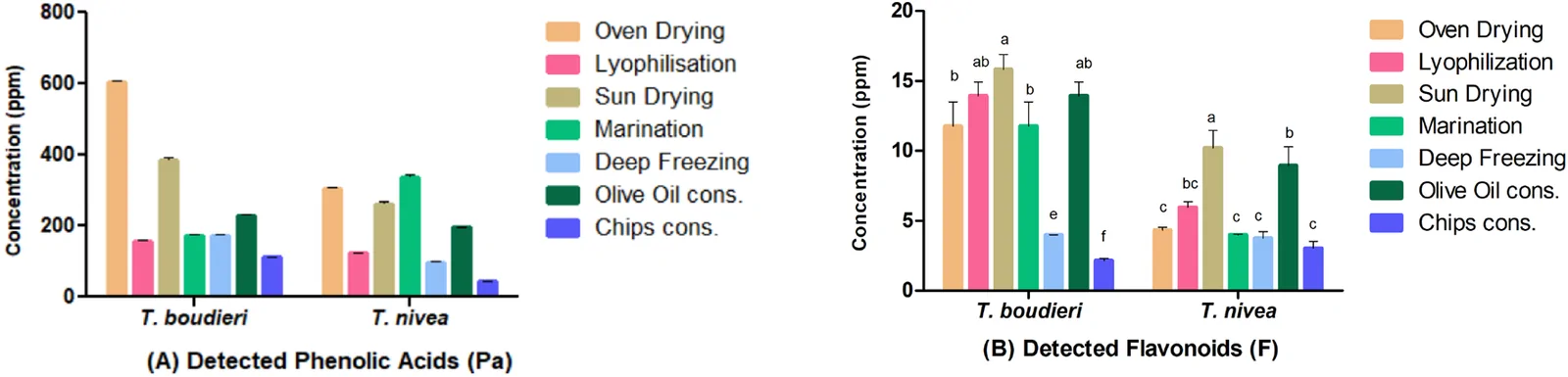
Qualitative bioactive compounds Phenolic acids **(A)** and flavonoids **(B)** in Terfezia boudieri and Tirmania nivea after seven conservation methods.

##### 4.3.3.2 Flavonoids (F)

Flavonoids (Rutin, Luteolin-7-o-glucoside, Naringin, Apegenin-7-o-glucoside, Quercetin, Kampherol, Naringenin, Apegenin, Luteolin, Cirsiliol and Cirsilineol) contents reveal that both species and conservation methods exerted significant effects on flavonoid content, with a significant interaction between factors (Interaction: 13.21%, P < 0.0001; Method: 57.75%, P < 0.0001; Species: 26.12%, P < 0.0001). Bonferroni post-hoc comparisons demonstrated that, relative to sun drying, oven drying and deep freezing substantially reduced flavonoid levels in both *T. boudieri* (−4.07 and −11.86; P < 0.001) and *T. nivea* (−5.87 and −6.46; P < 0.001), whereas lyophilization and olive oil conservation better preserved flavonoids (*T. boudieri*: −1.88, P < 0.05; *T. nivea*: −1.27, not significant). Conversely, chip processing and marination markedly decreased flavonoid content (*T. boudieri*: −13.66 and −4.07; *T. nivea*: −7.17 and −6.23; P < 0.001). Overall, *T. boudieri* exhibited higher flavonoid concentrations than *T. nivea*, and lyophilization or olive oil conservation emerged as the most effective strategies for flavonoid preservation.

## 5 Discussion

The present study clearly demonstrates that the conservation method is the principal determinant shaping the nutritional, mineral, vitamin, sugar, and phytochemical profiles of both truffle species, generally explaining most of the variability across parameters (often >70–95%), while species identity and interaction effects remain statistically significant but secondary. Overall, *T. boudieri* exhibited higher dry weight (≈18.9–23.0% vs. 15.3%–19.6%), fiber fractions (e.g., NDF up to 54.65%), organic matter, carbohydrates (up to 73.66 g/100 g DW), sodium, and flavonoids (15.83 vs. 10.20), whereas *T. nivea* showed higher ash content (up to 9.71 g/100 g DW), iron (≈1,283–1,600 ppm), magnesium, riboflavin (up to 3.94 ppm), and tocopherol. These differences likely reflect inherent physiological and ecological adaptations influencing nutrient allocation, mineral uptake, and metabolite storage.

Across all parameters, drying techniques, particularly oven drying and lyophilization, consistently enhanced measured concentrations of proteins (≈27–28%), phenolics (up to ≈142 mg GAE/g), antioxidant activity (≈87–89%), minerals, vitamins, sugars, and flavonoids, whereas chips processing generally produced the lowest values, including proteins (≈16%), minerals, sugars, and antioxidants. This overall increase under drying should be interpreted mainly as a concentration effect due to water removal, which increases the apparent content per gram of dry matter, but also as improved extractability of bound compounds after tissue structure disruption.

Lyophilization was the most efficient method for preserving and/or enhancing bioactive metabolites, as shown by its ability to maximize glucose (up to 6800 ppm), potassium (≈9,491 ppm), calcium (∼8,082 ppm), carotenoids, and phenolic acids. For example, phenolic acids reached 156.8–173.5 ppm in lyophilized samples compared with 383.4–605.4 ppm under sun and oven drying depending on species. This superiority is attributed to the low-temperature dehydration process, which minimizes thermal degradation, enzymatic oxidation, and structural collapse while maintaining cellular integrity and limiting loss of heat-sensitive metabolites.

Sun and oven drying showed intermediate effects but also clear evidence of thermal sensitivity for several metabolites. Flavonoids were significantly reduced under these treatments, with sun drying vs. oven drying showing decreases of −4.072 ppm (15.83 vs. 11.76) in *T. boudieri* and −5.872 ppm (10.20 vs. 4.330) in *T. nivea*. Similar trends were observed for antioxidant-related flavonoids, consistent with the fact that prolonged exposure to heat promotes oxidation and degradation reactions. Deep freezing preserved metabolites better than sun drying but remained less effective than lyophilization; for instance, phenolic acids decreased from 383.4 to 173.5 ppm in *T. boudieri* and from 262.7 to 96.46 in *T. nivea*, indicating that freezing maintains structure but may induce ice-crystal formation that affects cellular integrity and extractability.

The effects of marination observed in this study are consistent with recent literature describing acid-based preservation as a moderate process driven by osmotic exchange and diffusion phenomena. Immersion in acidic brine induces partial water loss while limiting complete dehydration, leading to intermediate dry matter levels as reported for osmotic and acidified food systems (Asghari et al., 2024; Giannakourou and Taoukis, 2021). In the present work, marination led to both decreases and occasional increases depending on species and metabolites. For example, phenolic acids showed reductions in *T. boudieri* (383.4 vs. 173.5 contextually compared to drying treatments), while *T. nivea* sometimes exhibited higher values (262.7 vs. 338.4), suggesting a balance between leaching losses and acid-induced solubilization or release of bound phenolics. The reduction in total phenolic content and flavonoids is mainly attributed to leaching of hydrophilic metabolites into the surrounding medium, a mechanism widely reported in immersed plant tissues (Kurhaluk et al., 2025). However, the acidic environment may partially limit oxidative degradation, which helps retain a fraction of phenolics and moderates overall losses. Vitamin and antioxidant responses are metabolite-dependent; water-soluble constituents are more susceptible to diffusion losses, whereas lipophilic metabolites are comparatively more stable in acidic systems (Darko et al., 2024). Accordingly, antioxidant capacity reflects a balance between phytochemical losses and partial stabilization under low-pH conditions (Gulcin, 2025).

The effects of chips processing and olive oil preservation on truffle lipid composition are closely related to the incorporation of exogenous oil during processing. In both treatments, contact with olive oil can modify the native lipid profile by increasing the proportion of unsaturated fatty acids and other lipophilic metabolites originating from the oil. This phenomenon is particularly evident during frying, where oil absorption occurs simultaneously with water loss, leading to an exchange between tissue moisture and external lipids (Valle et al., 2024). Similarly, prolonged storage in olive oil promotes diffusion of oil constituents into the truffle matrix, potentially altering both lipid composition and fatty acid distribution (Milani and Nemati, 2022). Despite this, crude fat contents remained lower in chips and olive-oil-preserved samples than in lyophilized or oven-dried truffles, suggesting that thermal degradation, oxidation of native lipids, and dilution effects associated with absorbed oil may partly offset the apparent increase in fat content. Furthermore, olive oil contributes antioxidant compounds such as phenolics and tocopherols, which can improve lipid stability during storage and reduce oxidative deterioration (Jimenez-Lopez et al., 2020). Therefore, the lipid fraction likely reflects a combination of native truffle lipids and absorbed olive-oil constituents.

These differences reflect the known impacts of preservation methods on water balance and tissue structure. Lyophilization removes water through sublimation under low temperature and vacuum conditions, minimizing cellular collapse and preserving structural integrity while concentrating dry matter constituents. Similarly, oven drying reduces moisture content through thermal evaporation, leading to an increase in the relative concentration of solids. The resulting reduction in water activity limits enzymatic reactions and microbial development while increasing the proportion of nutrients expressed on a dry-weight basis. The higher dry-weight values observed in *T. boudieri* may also be related to its greater carbohydrate and fiber contents, which contribute to solid matter accumulation and influence water-holding properties. These mechanisms collectively explain the higher dry matter levels recorded in dehydrated samples compared with marinated, oil-preserved, or frozen truffles.

The DPPH assay employed in this study is chemical analytical method that estimate the radical-scavenging capacity of extracts under *in vitro* conditions. While this assay is useful for comparing the relative antioxidant potential of samples and their phytochemical composition, it does not provide direct evidence of pharmacological efficacy or *in vivo* therapeutic antioxidant effects. Therefore, the present results should be interpreted solely as indicators of chemical antioxidant capacity.

These results confirm and extend previous research showing that post-harvest processing is a stronger determinant of nutritional and bioactive composition than species identity in edible fungi. Similar to our finding that drying explains most of the variability across parameters, studies on desert truffles and mushrooms have shown that dehydration methods strongly influence phenolic content, antioxidant activity, vitamins, and minerals more than genetic differences between taxa (Villalobos-Pezos et al., 2025; Paliwal et al., 2023; Murcia et al., 2002). Specifically, the superiority of freeze-drying in preserving sugars, minerals, phenolics, and antioxidants is consistent with reports in mushrooms and food products where lyophilization retained higher bioactive and nutrient levels than sun or hot-air drying (Koffi et al., 2024; Moyo, 2024). The observed increases in measured protein and antioxidant effectiveness after oven or freeze-drying align with findings that water removal concentrates nutrients and can enhance extractability of bound phenolics (Thomas et al., 2025; Koffi et al., 2024). Our inter-species differences, with *T. boudieri* richer in carbohydrates and fiber and *T. nivea* higher in ash, iron, and vitamins, parallel compositional surveys showing that *Terfezia spp.* tend to accumulate polysaccharides, while *Tirmania spp.* exhibit elevated mineral and vitamin profiles. The influence of processing medium, such as marination or oil preservation affecting mineral and carotenoid retention, has also been documented in edible mushrooms, where acidic or lipid environments modify extractability and stability of specific metabolites (Barros et al., 2008; Rozsa et al., 2017).

Overall, preservation strategy emerges as the dominant determinant of truffle compositional quality, with drying methods generally optimizing nutritional and bioactive retention, while species traits modulate the magnitude and direction of these effects. The differences observed among conservation methods can be attributed to their distinct effects on biochemical stability, water activity, and metabolite solubility. Lyophilization consistently resulted in higher retention of bioactive metabolites due to low-temperature dehydration that minimizes thermal degradation and enzymatic activity, while also concentrating solutes per gram of dry matter. In contrast, conventional thermal drying may induce partial degradation of heat-sensitive compounds due to prolonged exposure to elevated temperatures. Deep freezing limits enzymatic activity but may still allow structural damage through ice crystal formation, affecting compound or metabolite extractability. Marination and olive oil preservation introduce diffusion and partitioning phenomena that modify the distribution of hydrophilic and lipophilic metabolites, while chips conservation showed the lowest values in several cases, likely due to combined processing losses and leaching effects.

## 6 Conclusion

This study evaluated the impact of seven conservation methods (sun drying, oven drying, lyophilization, deep freezing, chips processing, marination, and olive-oil preservation) on the nutritional and phytochemical quality of *T. boudieri* and *T. nivea*. The results demonstrated that conservation method was the principal factor driving variability in proximate composition, minerals, vitamins, sugars, and bioactive metabolites, while species differences were secondary. Overall, drying techniques, particularly lyophilization and oven drying, provided the best preservation of nutritional and bioactive quality due to efficient moisture removal and reduced enzymatic and oxidative degradation, with lyophilization emerging as the most effective method for maintaining heat-sensitive and bioactive metabolites through superior structural preservation. In contrast, chips processing and marination led to the greatest losses, mainly due to leaching, diffusion into surrounding media, and processing-induced degradation. Deep freezing showed intermediate effectiveness by limiting enzymatic activity but inducing structural alterations that affected metabolite stability. Olive-oil preservation exhibited dual effects, improving stability of certain metabolites through antioxidant transfer while simultaneously modifying the native chemical profile due to lipid incorporation. Species-related differences were consistent but less influential than processing effects, with *T. boudieri* generally richer in carbohydrates and fiber, and *T. nivea* characterized by higher mineral and vitamin contents. Overall, lyophilization appears to be the most suitable preservation method, followed by controlled oven drying and deep freezing, whereas marination and chips processing are less favorable for preserving nutritional and bioactive integrity. These findings highlight the critical importance of selecting appropriate post-harvest preservation strategies to maintain the functional value of desert truffles.

## Data Availability

The original contributions presented in the study are included in the article/supplementary material, further inquiries can be directed to the corresponding author.
